# Wine Phenolic Compounds Differently Affect the Host-Killing Activity of Two Lytic Bacteriophages Infecting the Lactic Acid Bacterium *Oenococcus oeni*

**DOI:** 10.3390/v12111316

**Published:** 2020-11-17

**Authors:** Cécile Philippe, Amel Chaïb, Fety Jaomanjaka, Stéphanie Cluzet, Aurélie Lagarde, Patricia Ballestra, Alain Decendit, Mélina Petrel, Olivier Claisse, Adeline Goulet, Christian Cambillau, Claire Le Marrec

**Affiliations:** 1 EA4577-USC1366 INRAE, Unité de Recherche OEnologie, Université de Bordeaux, Institut des Sciences de la Vigne et du Vin (ISVV), F-33140 Villenave d’Ornon, France; cecilemphilippe@gmail.com (C.P.); bounoise68@hotmail.fr (A.C.); jaomazava@yahoo.fr (F.J.); stephanie.cluzet@u-bordeaux.fr (S.C.); aurelie.lagarde@u-bordeaux.fr (A.L.); patricia.ballestra@u-bordeaux.fr (P.B.); alain.decendit@u-bordeaux.fr (A.D.); olivier.claisse@u-bordeaux.fr (O.C.); 2 Bordeaux Imaging Center, UMS3420 CNRS-INSERM, University Bordeaux, F-33000 Bordeaux, France; melina.petrel@u-bordeaux.fr; 3 INRAE, ISVV, USC 1366 Oenologie, F-33140 Villenave d’Ornon, France; 4 Architecture et Fonction des Macromolécules Biologiques, Aix-Marseille Université, Campus de Luminy, F-13020 Marseille, France; adeline.goulet@univ-amu.fr (A.G.); ccambillau@gmail.com (C.C.); 5 Architecture et Fonction des Macromolécules Biologiques, Centre National de la Recherche Scientifique (CNRS), Campus de Luminy, F-13020 Marseille, France; 6 Bordeaux INP, ISVV, EA4577 OEnologie, F-33140 Villenave d’Ornon, France

**Keywords:** bacteriophage, predation, polyphenols, receptor-binding protein, adsorption

## Abstract

To provide insights into phage-host interactions during winemaking, we assessed whether phenolic compounds modulate the phage predation of *Oenococcus oeni*. Centrifugal partition chromatography was used to fractionate the phenolic compounds of a model red wine. The ability of lytic oenophage OE33PA to kill its host was reduced in the presence of two collected fractions in which we identified five compounds. Three, namely, quercetin, myricetin and *p*-coumaric acid, significantly reduced the phage predation of *O. oeni* when provided as individual pure molecules, as also did other structurally related compounds such as cinnamic acid. Their presence was correlated with a reduced adsorption rate of phage OE33PA on its host. Strikingly, none of the identified compounds affected the killing activity of the distantly related lytic phage Vinitor162. OE33PA and Vinitor162 were shown to exhibit different entry mechanisms to penetrate into bacterial cells. We propose that ligand-receptor interactions that mediate phage adsorption to the cell surface are diverse in *O. oeni* and are subject to differential interference by phenolic compounds. Their presence did not induce any modifications in the cell surface as visualized by TEM. Interestingly, docking analyses suggest that quercetin and cinnamic acid may interact with the tail of OE33PA and compete with host recognition.

## 1. Introduction

After alcoholic fermentation, most wines undergo malolactic fermentation (MLF), driven by the lactic acid bacterium *Oenococcus oeni*, which improves their organoleptic properties and microbiological stability. Wine is a temporally dynamic multi-stressor environment for lactic acid bacteria (LAB) and other microorganisms. Amongst the prominent constraints experienced by wine bacteria, the exposure to phenolic compounds (PCs) is one of the most complex to apprehend [1,2,3,4]. Wine contains a vast diversity of PCs, most of which originate in the grape berry. They are important quality components that contribute to essential organoleptic properties including color, astringency and bitterness. The total phenolic content varies among major wine-producing countries and is usually about 1.5 g L^−1^ in red wines while white wine contains only 100–400 mg L^−1^ [5].

The growth and viability of wine-related microorganisms are affected by PCs in different ways depending on their type, concentrations and environment [6,7,8]. The concentrations of the major PCs in wines are significantly lower than the minimum inhibitory concentration values established against wine LAB [2]. It is therefore unlikely that a PC alone may affect the growth of LAB at the concentrations found in wine. However, both additive and synergistic effects among all antimicrobial present PCs (and likely other stressors) may promote the inactivation of LAB in the wine environment. As a matter of fact, a significant inhibitory impact on undesirable bacteria can be obtained through the enrichment of wines with PCs [8]. The strategy has been considered as an alternative to the use of sulfur dioxide, one of the most versatile and efficient additives used in winemaking to limit spoilage bacteria and control MLF [9].

The lethal action of PCs on bacteria has been attributed to multiple mechanisms and different components of the cell envelope are well-established targets [10]. Detrimental changes in the bacterial cell ultrastructure occur including an increase in thickness, the presence of fluffy polysaccharides on the cell surface and cell aggregation or disruption [11,12]. Interactions with membranes also play an essential role in the antibacterial activity of many PCs and flavonoids are known to partition into lipid membranes and reduce their fluidity [13]. Lethality also results from the ability of PCs to inhibit critical enzymes involved in cell wall, nucleic acid and ATP synthesis [10].

Beyond the scope of enology, new insights have been gained over the last few years about the interactions between LAB and sublethal PC concentrations in microbial ecosystems such as plant-based fermentations and the human gut. PC concentrations that do not impair cell growth were shown to modulate the interactions of bacteria with the extracellular environment and their capacity to adhere to abiotic and biotic surfaces [14,15,16]. When added as pure molecules, several PCs could induce proteome and transcriptome changes in probiotic LAB as well as commensal gut bacteria with significant effects on protein folding, cell wall synthesis and the production of exopolysaccharide and surface proteins [15,16,17,18]. Other authors also reported that bacterial cell surface alteration by PCs can modulate phage-host interactions in *Lactobacillus casei* [19] and more recently in *Staphylococcus aureus* [20]. Taking advantage of the recent isolation of lytic phages OE33PA [21] and Vinitor [22,23] infecting *O. oeni*, we explored the hypothesis that phage-host interactions may vary in the presence or absence of PCs. We identified a set of molecules that decreased the ability of phage OE33PA to kill *O. oeni* while predation by the distantly related lytic phage Vinitor162 was not impaired. The isolation of bacterial insensitive mutants and comparisons of the structure of the host recognition devices are consistent with the recognition of distinct receptors on the host cell surface by OE33PA and Vinitor162. Our current work suggests that PCs may be indirect drivers of bacterial diversity through their differential modulation of phage predation of *O. oeni* in the enological environment. Possible interactions of PCs with an OE33PA host recognition device, which could affect phage adsorption to the surface of *O. oeni*, are discussed.

## 2. Materials and Methods

### 2.1. Preparation of Phenolic-Enriched Extracts from a Red Wine by Centrifugal Partition Chromatography

A Cabernet Sauvignon red wine from the south of France (Vieux Carion Pays d’Oc, 2012) was used for this study. A volume of one liter was concentrated down to 200 mL in vacuo at 30 °C. After sonication, the concentrated wine was poured over 500× *g* of Amberlite XAD-16 resin (Sigma-Aldrich, St. Louis, MO, USA), in an open column (5.1 cm × 35 cm). We then added 5 L of water by 500 mL steps in order to remove sugars, small organic acids and other non-phenolic molecules. PCs were eluted with 2 L of pure grade acetone (Xilab, Bruges, France). After evaporation of the solvent in vacuo, the phenolic-rich extract was lyophilized.

The phenolic-rich extract was fractioned by centrifugal partition chromatography (CPC) using a FCPC200^®^ apparatus of 200 mL capacity (Kromaton Technologies, Sainte-Gemmes-sur-Loire, France), connected to a Gilson 321-H1 binary pump with a 20 mL sample loop. The ARIZONA system D (n-heptane-EtOAc-MeOH-H_2_O, 1-6-1-6) was chosen. HPLC grade ethyl acetate (EtOAc, Scharlau, Barcelona, Spain) and methanol (MeOH, Carlo Erba, Rodano, Italy) as well as HPLC grade n-heptane (Sigma-Aldrich, St. Louis, MO, USA) were used. Water was purified using an Elga water purification system (Bucks, UK). A protocol described earlier for the extraction of wine PCs [24] was used with slight modifications. Two grams of the lyophilized phenolic-rich extract were reconstituted with 12 and 6 mL of the upper and lower phases, respectively. It was then sonicated (5 min) and filtered (0.45 µm). The column was filled with 600 mL of the stationary (lower) phase starting at 0 rpm for the first 200 mL and 500 rpm for the last 400 mL. The mobile phase (upper phase) was then added at 3 mL min^−1^ giving a dead volume of approximately 70 mL. The CPC fractions were monitored by a UV detector. At the end of 180 min of the ascending mode, the column was switched to descending mode and ran for an additional 120 min. One hundred and two fractions were collected. After 300 min, MeOH/H_2_O (50/50) was added and two more CPC fractions were obtained. Samples of all fractions were kept at −20 °C. All fractions (15 µL) were then loaded on silica plates (Magerey-Nagel, Hoerdt, France) with a Camag Automatic TLC Sampler III (Chromacim SAS, Moirans, France) and the compounds were run in a chloroform/MeOH/acetic acid (85/15/3) solvent. The plates were observed under visible and UV (254 and 366 nm) light. Fractions displaying similar phenolic profiles on a thin layer chromatogram (TLC) were pooled, which yielded 32 fractions. They were evaporated to dryness and dissolved in ethanol (Merck KGaA, Darmstadt, Germany) for the CPC fractions previously obtained in the ascending mode or in MeOH/acetone (75/25) for fractions derived from the descending mode.

### 2.2. Determination of Total Phenols of Fractions

The total phenolic content was determined by the Folin–Ciocalteu colorimetric method [25] adapted to a 96-well plate. The fractions were diluted 10-, 50- or 100-fold in methanol. To 25 µL of diluted extract or methanol (blank), 125 µL of Folin–Ciocalteu’s reagent (Sigma-Aldrich, St. Louis, MO, USA) (diluted 10 times with H_2_O) were added. After 2 min, 100 µL of sodium carbonate (75 g L^−1^) solution (Sigma-Aldrich, St. Louis, MO, USA) was added. The mixture was then kept in the dark at room temperature for 1 h. The absorbance was measured at 765 nm using a plate reader (Fluostar Optima; BMG Labtech, Champigny-Sur-Marne, France). The total phenolic content was expressed as mg gallic acid equivalent (GAE) per g of recovering solvent (mg GAE mL^−1^). The samples were analyzed in triplicate.

### 2.3. Identification of Phenolic Compounds in the Biologically Active Fractions

Two biologically active fractions, named F9 and F11, were analyzed by HPLC with an Agilent 1200 (Agilent Technologies, Santa Clara, CA, USA) and an Agilent spectrometer UV-visible-DAD detector. A Prontosil C18 (5 mm × 150 mm; 4.6 µm) column was used (Bischoff, Leonberg, Germany). Both fractions were dissolved in MeOH:H_2_O (50:50; 1.5 mg mL^−1^), filtered (0.45 µm) and injected using a 1 µL injection volume. The fractions were eluted with a gradient consisting of H_2_O acidified with 0.1% formic acid (solvent A) and acetonitrile (ACN) acidified with 0.1% formic acid (solvent B) at 1 mL min^−1^. LC–MS grade ACN (Scharlau, Barcelona, Spain) and formic acid (Fischer Scientific, Loughborough, UK) were used. A 60 min elution was performed with a gradient of 0 min (95% A, 5% B) to 50 min (65% A, 35% B) followed by a 5 min wash (100% B) and a 10 min re-equilibration step. The column effluent was monitored at 25 °C in the range 190–400 nm with the acquisition of the full spectra. Four different wavelengths (280, 306, 320 and 360 nm) were selected in order to determine the optimal absorption range for trans- and cis-stilbenes, phenolic acids and flavonoids, respectively. The HPLC was coupled to an Esquire 3000 Plus ion trap mass spectrometer (Bruker Daltonics, Billerica, MA, USA) using an ESI source (Agilent Technologies). The chromatographic conditions were as mentioned above and the HPLC output was split 1:10 into the MS detector. The total ion chromatograms were obtained using a positive mode with a range of m/z 110–1500. Nitrogen was used as the drying gas at 5 L min^−1^ with a nebulizer pressure of 15 psi at 325 °C. For the negative ion mode, the capillary voltage was −3700 V, the capillary end voltage was at 127.7 V, the skimmer voltage was at 40 V and the trap drive was at 68.7. Data analysis was performed with Bruker Data Analysis 3.2 software (Palaiseau, France).

The identification of the compounds was based on chromatographic behavior (retention times, UV-Vis absorption maxima) and mass spectra obtained in a positive mode by using their fragmentation patterns and literature data: trans- and cis-resveratrol [26,27]; *p*-coumaric acid, quercetin and myricetin [28,29,30].

### 2.4. Phage and Strains

OE33PA is an ex-temperate phage. This virulent mutant harbors an IntB-type integrase sequence [21,31]. Vinitor162 is a professional lytic phage [22,23]. These two phages were previously isolated from red and sweet white wines, respectively. The host bacterium, *O. oeni* IOEBS277, was grown in liquid or solid MRS (Man Rogosa Sharpe) (Difco, Fischer Bioblock Scientific, Illkirch, France) adjusted to pH 4.8 at 25 °C. The propagation of OE33PA was carried out in MRS_Φ_ (MRS supplemented with MgSO_4_ 3.75 g L^−1^ and CaCl_2_ 2.375 g L^−1^) [32]. The phage was added to exponential phase bacterial cultures (OD_600_ 0.1) with a ratio of phages added to the bacteria (multiplicity of infection or MOI) of 0.003. The infection was assessed by OD_600 nm_ measurement. The phages were enumerated on the indicator strain *O. oeni* IOEBS277 using the classical double-layer plating technique on MRS_Φ_ agar [32]. The plates were incubated at 25 °C for four to seven days before the examination of plaques. The phage counts were expressed as plaque-forming units per mL (PFU mL^−1^).

### 2.5. Killing-Curve Assays with the CPC Fractions

A culture of *O. oeni* IOEBS277 (OD_600 nm_ 0.15) was divided into two portions of 1 mL each. Phenolic compounds were added as CPC fractions at a rate of 10% *v*/*v* in the first portion (assay), which did not modify the pH (4.8 ± 0.05). The second portion was added with 10% *v*/*v* of ethanol, the solvent used to resuspend the fraction tested (control). Each culture (assay and control) was subdivided into two portions and the phage was added to one of them to a final MOI of 0.003 as determined by plaque forming unit (PFU) and colony forming unit (CFU) plating. The four cultures (assays infected (Ai) or not (Ani) and controls infected (Ci) or not (Cni)) were then transferred to a 96-well plate (200 µL/well) and incubated in a microplate incubator (Synergy HT, BIO-TEK, Colmar, France) at 30 °C during 35–40 h with OD_600 nm_ measures recorded every 12 h. The values corresponding to the differences between Ani and Ai and Cni and Ci, respectively, were compared.

Instead of CPC-bioactive fractions, pure commercial phenolic molecules were then tested. All were purchased from Sigma-Aldrich (St Louis, MO, USA) and prepared as concentrated solutions (5 mg mL^−1^) in ethanol.

The subinhibitory concentrations that did not affect bacterial growth in MRS broth nor the stability of both phage lysates were first determined. To this aim, each compound was added at various final concentrations (0–100 µg mL^−1^) to a bacterial culture (OD_600 nm_ 0.15) in MRS broth. The samples were withdrawn over 72 h and CFU were enumerated on MRS agar plates. Phage lysates (10^7^ to 10^8^ PFU mL^−1^) and dilutions thereof were also incubated for 72 h in MRS in the presence of each compound and PFU were enumerated as described before. The final concentrations of phenolic molecules having no substantial change in bacterial counts in the non-infected culture nor in the phage titer in the lysates after 72 h of incubation were selected for the killing-curve assays.

### 2.6. Killing-Curve Assays with Pure Molecules

For each selected compound, 10 mL cultures (Ani, Ai, Cni and Ci) were prepared as mentioned above. All of the experiments were done in triplicate. Samples (1 mL) were periodically collected and centrifuged. The PFU in the supernatant were enumerated. The cell pellet was washed and resuspended in 1 mL of MRS broth. The PFU were determined as described before.

### 2.7. Adsorption Assays

Pre-cultures of *O. oeni* IOEBS277 were grown in MRS with or without quercetin (Q) or morin (M) (50 µg mL^−1^) (MRS+Q or MRS+M) to an OD_600 nm_ of 1. The cells were next diluted in the same media to OD_600 nm_ 0.1. When OD_600 nm_ 0.2 was reached, a volume of 1 mL of each culture was centrifuged and cells were resuspended in 900 µL of fresh warm MRS+Q or MRS+M broth (25 °C). A volume of 100 µL of diluted phage lysate was added in order to yield an MOI of 0.003. The mixture was incubated at 25 °C to allow phage adsorption. The samples were withdrawn at T0 and after 20 and 40 min, filtered through a 0.45 µm filter membrane and serially diluted in MRS_Φ_ broth. The free phage titer (unbound phages) in the samples was determined by counting the PFUs. The results at 20 and 40 min were expressed as a percentage to the initial phage count. The experiment was replicated three times.

### 2.8. Isolation of Phage-Insensitive Mutants

Bacteriophage-insensitive mutants (BIM) against OE33PA and Vinitor162 phages were obtained as follows. An exponentially growing culture of the sensitive strain *O. oeni* IOEBS277 (OD_600 nm_ 0.2) in MRS_Φ_ was infected with OE33PA or Vinitor162 with an MOI of 0.01 and incubated at 25 °C. Complete lysis occurred within two days in both samples. Upon prolonged incubation, regrowth was observed at day nine and cultures were streaked on grape juice agar plates at day twelve. After five days of incubation at 25 °C, ten colonies from each sample were isolated and purified by three consecutive streakings on MRS agar. They were recorded as presumptive BIMs and named BIM_OE33PA_ and BIM_Vinitor162_, respectively. The efficiency of plaquing (EOP), defined as the ratio between the PFU mL^−1^ obtained on each BIM and the PFU mL^−1^ obtained on the parent strain (IOEBS277) in double-layer plaque titrations, was investigated on each BIM as a marker of their resistance level against phage OE33PA and/or Vinitor162 infection. A value < 10^−6^ indicated that the strain was resistant to the challenge phage.

All confirmed BIM clones were analyzed by the variable number of tandem repeats typing method (VNTR) [33] to demonstrate that they were derivatives of strain IOEBS277. The absence of signals with primers designed on the distinct integrase sequences reported for oenophages was checked, showing that resistance of the BIM_OE33PA_ isolates was not linked to any accidental lysogeny or pseudo-lysogeny event during the manipulations.

### 2.9. Transmission Electron Microscopy

Bacterial cells grown overnight in the absence or presence of PCs (50 µg mL^−1^) were prepared and stained according to Thiery’s silver proteinate staining method to visualize polysaccharides [34,35]. It uses the oxidation of polysaccharides by periodic acid to create aldehyde groups, which are visualized by a silver complex. Ultra-thin sections were examined with a Hitachi H7650 electron microscope operated at 80 kV as previously described [35].

### 2.10. Homology Modeling of OE33PA and Cinnamic Acid and Quercetin Docking

The homology model of the OE33PA receptor-binding protein (RBP) head domain was built using MODELLER in the MPI Bioinformatics Toolkit [36] and the phage p2 RBP crystal structure was the template (PDB ID 1ZRU). We obtained the cinnamic acid and the quercetin 3D structures from the protein data bank (PDB) entries 4cq5 and 1h1i, respectively (named TCA and QUE, respectively) and docked them as rigid bodies into the receptor-binding pockets of the OE33PA RBP homology model using Coot [37,38].

## 3. Results

### 3.1. Influence of Phenolic Compounds on the Lytic Development of Phage OE33PA on Its Host

A bioguided fractionation of a Cabernet Sauvignon red wine with centrifugal partition chromatography yielded 32 main fractions (F1 to F32) with total phenolic contents ranging from 10 to 100 mg GAE mL^−1^. The potential of each main fraction to influence the lytic development of oenophage OE33PA on its host *O. oeni* IOEBS277 was further tested in MRS_Φ_ broth over 48 h.

**Figure 1:**
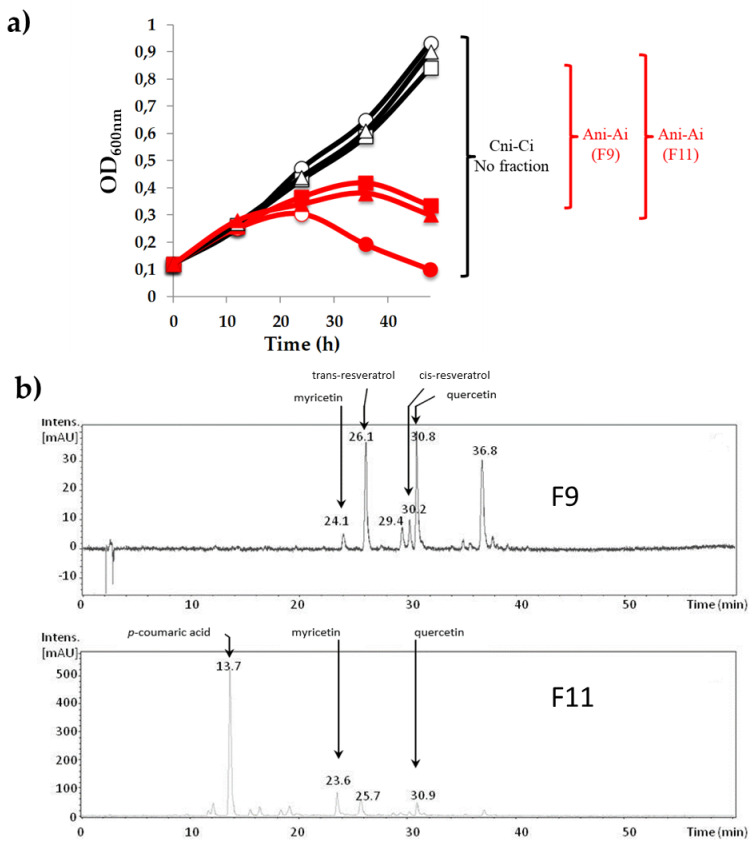
Phenolic fractions F9 and F11 reduce the killing activity of phage OE33PA on *Oenococcus oeni* in Man Rogosa Sharpe (MRS_Φ_) broth. (**a**) black curves correspond to the three non-infected (ni) cultures: the control culture without fraction (Cni, ◯) and assays with 10 µl of fraction 9 (F9) (Ani, □) or fraction 11 (F11) (Ani, △). Red curves correspond to the three cultures infected with phage OE33PA with a multiplicity of infection (MOI) of 0.003, the control without fraction infected (Ci, ●) and infected assays (Ai) with fractions 9 (■) or 11 (▲). Fractions F9 and F11 contained 7.95 ± 2.14 and 74.32 ± 1.98 mg GAE mL^−1^, respectively. (**b**) LC chromatograms at 306 nm of the F9 and F11 fractions. Peak identification was based on (i) analysis of the retention times (values are given), wavelengths of maximum absorbance (λmax), pronated molecules ([M+H]^+^) and major fragment ions (MS/MS in positive mode) and (ii) comparison with published literature [28,29,30].

This preliminary analysis showed that host cell lysis due to phage lytic propagation was reduced in the presence of fraction F9 or F11 (10%) compared with the infected control cultures (Figure 1a). None of the tested F9 and F11 fractions inhibited bacterial growth in the absence of phage. It was therefore suggested that fractions F9 and F11 may inhibit the bacterial killing activity of phage OE33PA and protect the bacterial cells from phage predation.

The major PCs contained in both biologically active fractions were identified using a reverse phase HPLC system coupled with an MS (Figure 1b). Fractions F9 and F11 showed the presence of 9 and 14 peaks, respectively. Two peaks were common to both fractions and corresponded to the flavonoids quercetin and myricetin. Two additional peaks from fraction F9 were assigned to trans- and cis-resveratrol (stilbenoids), one remained undetermined and one peak from fraction F11 could be identified as *p*-coumaric acid (phenolic acid) (Figure 1b and Table S1).

**Figure 2:**
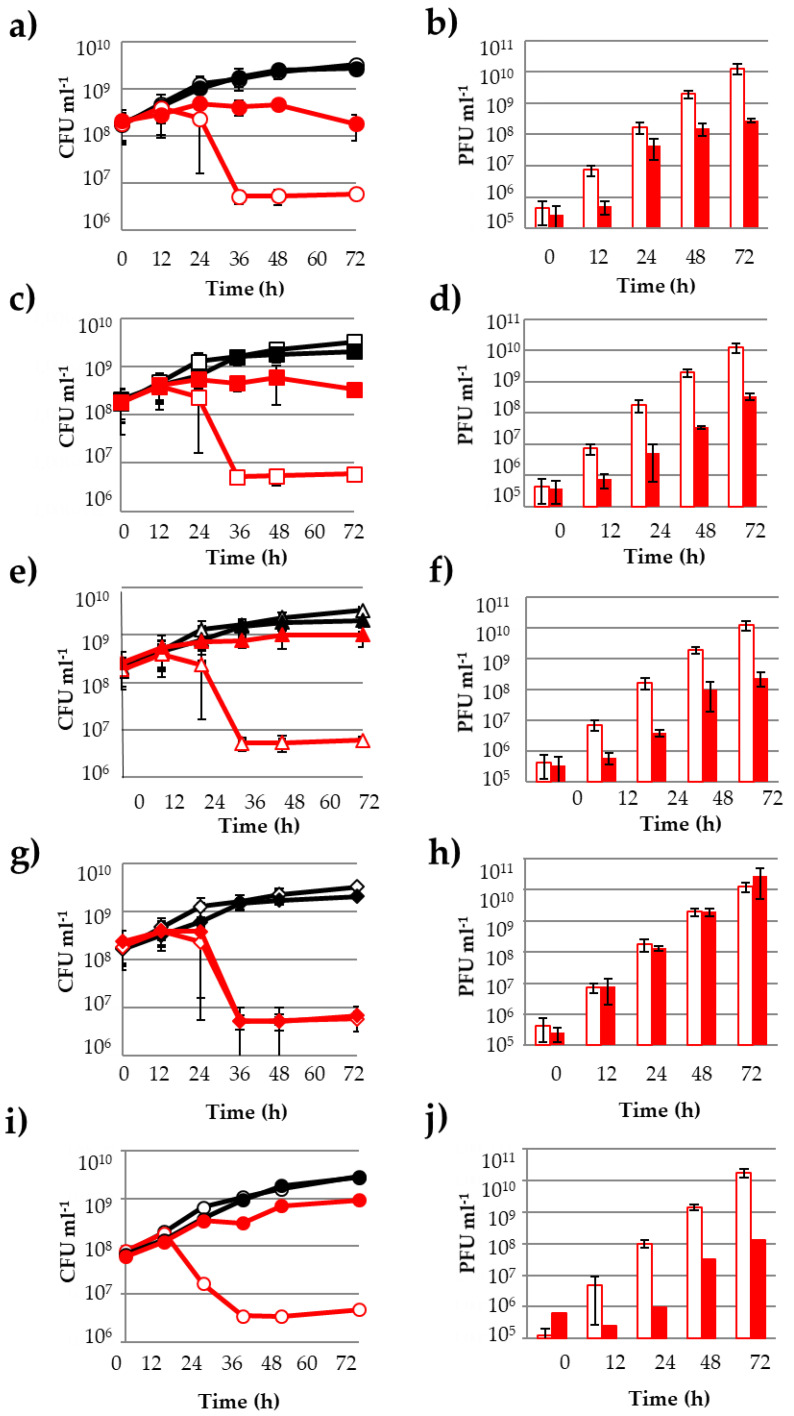
The effect of pure phenolic compounds on the infection cycle of OE33PA on *O. oeni* IOEBS277. (**a**,**b**) *p*-coumaric acid; (**c**,**d**) quercetin; **(e**,**f**) myricetin; (**g**,**h**) trans-resveratrol (cis-resveratrol yielded similar results) and (**i**,**j**) morin. Infected and non-infected cultures are in red and black, respectively. Controls without phenolic compounds are indicated with an empty mark. Filled marks represent cultures where 50 µg mL^−1^ of phenolic compounds were added. (**a**,**c**,**e**,**g**,**i**) represent bacterial growth curves (CFU mL^−1^) and (**b**,**d**,**f**,**h**,**j**) represent free phages in the supernatant (PFU mL^−1^). Experiments were done in triplicate.

All five putative bioactive PCs (*p*-coumaric acid, quercetin, myricetin, trans- and cis-resveratrol) were purchased as pure compounds and further tested for their ability to reduce OE33PA-mediated bacterial cell lysis over 72 h (Figure 2). The concentration of 50 µg mL^−1^ was used for each compound as it did not affect the growth of *O. oeni* in MRS nor the infectivity of a high titer lysate of phage OE33PA over a 72 h incubation period (Figure S1). We first compared the growth of the bacterium in the presence and absence of the phage in the medium deprived of PCs. The growth of *O. oeni* IOEBS277 was inhibited by OE33PA at an early stage of growth. Infection produced a decline of the population with an approximately 2-log drop in CFUs between 12 and 36 h and the release of new phage particles reaching a final concentration of 10^10^ PFU mL^−1^ (Figure 2). No bacterial regrowth occurred in the course of our experiment, which is in agreement with the strictly lytic development of phage OE33PA on its host. In the presence of *p*-coumaric acid (Figure 2a), quercetin (Figure 2c) or myricetin (Figure 2e), OE33PA had a reduced impact on the control of bacterial densities. Bacterial counts showed little or no reduction in the infected cultures over time and populations remained above 10^8^ CFU mL^−1^. Phage counts also confirmed the reduced release of progeny phages in the supernatants of PC-treated samples compared with the infected control culture (Figure 2b,d,f). Greater than 1.5-log reductions in phage levels were observed in PC-treated infected cultures compared with untreated infected cultures, suggesting that OE33PA was less effective in the presence of the tested molecules. In contrast, when trans- or cis-resveratrol was added, bacterial counts and phage titers in the supernatant over infection were both similar compared with the untreated and infected cultures, showing that these two molecules did not modify the dynamics of phage infection (Figure 2g,h).

Thus, the reduction of the killing activity of OE33PA observed for F9 and F11 could result from the presence of quercetin (F9 and F11), myricetin (F9 and F11) or *p*-coumaric acid (F11). We cannot at this stage rule out the possibility that other uncharacterized compounds from both CPC red wine fractions may also have such an activity on phage propagation.

### 3.2. Other PCs Possess the Ability to Impair Phage Lytic Cycle on O. oeni

Other PCs structurally related to *p*-coumaric acid, quercetin and myricetin and differing in chemical structures and functional groups were selected and tested (Table 1).

Cinnamic acid and caffeic acid exhibited the same inhibitory properties previously shown for *p*-coumaric acid. The situation was more complex with flavonoids. In addition to quercetin and myricetin, we also tested morin and kaempferol. All four flavonols share the C15 skeleton of flavones with only hydroxyl groups as substituents on the three rings (A, B and C) (Table 1). Surprisingly, morin was shown to reduce phage killing (Figure 2i,j) while kaempferol was not (Table 1). Interestingly, morin and kaempferol differ in the specific position of a single hydroxyl group. Retusin, a flavonol characterized by the presence of a methoxyl group in four positions (on the A, B and C rings) was then tested and also observed to inhibit phage development [39]. As wine contains considerable amounts of flavonol glycosides, we chose to test one of the main compounds, namely, quercetin-3-*O*-glucuronide. We observed that the glycosylated flavonoid did not have the property described for quercetin. We also observed no effect for piceid, a glycosylated form of resveratrol. As a conclusion, we identified seven PCs (four flavonols and three phenolic acids) that possessed the ability to impair the phage OE33PA lytic cycle on *O. oeni* with quercetin, myricetin and morin being the most inhibitory to phage propagation. Our data suggest that the bioactivity of PCs observed in this study may vary according to different features such as the hydrophily, the orientation of aromatic rings and/or the specific distribution of functional groups on the molecules.

### 3.3. Adsorption of Phage OE33PA Is Impaired by Phenolic Compounds

To shed light on the inhibitory mechanisms of the bioactive PCs identified in our study, we next tested the idea that OE33PA performs less efficiently in the presence of these molecules due to reduced adsorption to bacterial cells. Bacterial cells were grown for 24 h in MRS broth in the presence (MRS+Q) or absence of quercetin (MRS). Cells were collected and resuspended in the same media (MRS or MRS+Q). Adsorption assays were performed to identify the rate at which phage OE33PA adsorbed to the surface of *O. oeni* in both conditions (initial concentrations of bacteria and phage were ~2 × 10^8^ and 0.6 × 10^6^ PFU mL^−1^, respectively).

**Figure 3:**
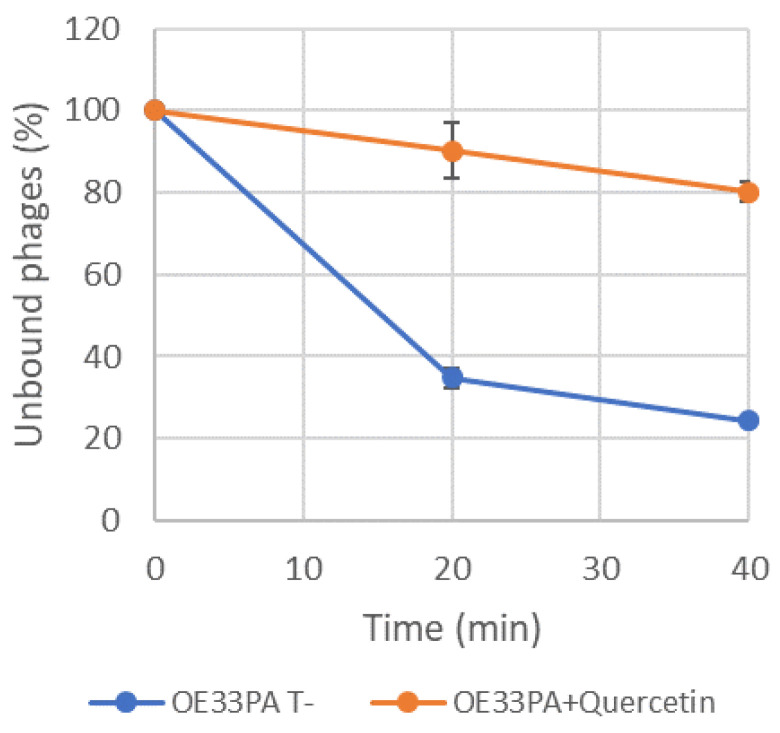
Adsorption assay of phage OE33PA on *O. oeni* IOEBS277. Cells were grown in MRS in the absence (blue) or presence of quercetin (50 µg mL^−1^) (orange). The test was done in the same media and infection was carried out at an MOI of 0.003.

The resulting graph (Figure 3) revealed a rapid decrease in free phage concentration over time in the control with around 20% of the phages unbound after 40 min. In contrast, phage OE33PA had a severely reduced adsorption efficiency on cells grown in the presence of quercetin for 24 h and 80% of the phages were not adsorbed after 40 min. In our experimental set up, the adsorption of phage OE33PA on the cell wall was impaired in the presence of quercetin. Similar data were obtained with morin [40].

### 3.4. PCs Do Not Affect the Host-Killing Activity of Vinitor162 on O. oeni

We next sought to determine whether quercetin could modulate the killing activity of other lytic oenophages. Vinitor162, a representative of the recently characterized Vinitor group, was chosen. This phage is strictly lytic and distantly related to temperate oenophages [22,23]. Experiments with Vinitor162 were conducted at an MOI of 0.003 and run in parallel with an assay with phage OE33PA. In untreated cultures, Vinitor162 could lyse *O. oeni* after 20 h of incubation. However, its killing activity was not modified in the presence of any of the seven phenolic compounds active on the predation of *O. oeni* IOEBS277 by OE33PA (Figure S2, Table 1). Similar data were observed when higher and lower MOI were tested (data not shown).

### 3.5. Phages’ Adhesion Devices: *Homology* Detection and Structure *Prediction*

Taken together, our findings suggest that the seven PCs identified in our study could inhibit the lytic propagation of OE33PA through an interference with its adsorption to the host cell wall. In contrast, no PC-induced inhibition occurred in the presence of the distinct phage Vinitor162. This prompted us to better characterize the lytic propagation of these two oenophages on their common host and assess whether host adsorption engaged different devices (phage receptor-binding protein, or RBP, and its bacterial receptor). We first conducted single high titer phage challenges of *O. oeni* IOEBS277 with phage OE33PA or Vinitor162 in order to select bacteriophage-insensitive mutants (BIM). BIM are typically achieved through mutations affecting bacterial-surface molecules (adsorption resistance), thereby interfering especially with phage attachment. The challenge of *O. oeni* with OE33PA resulted in the emergence of BIM, which remained sensitive to Vinitor162. Likewise, BIM obtained after challenging the sensitive strain with Vinitor162 were still sensitive to OE33PA. Hence our data suggest that OE33PA and Vinitor162 recognized distinct bacterial cell wall structure components.

**Figure 4:**
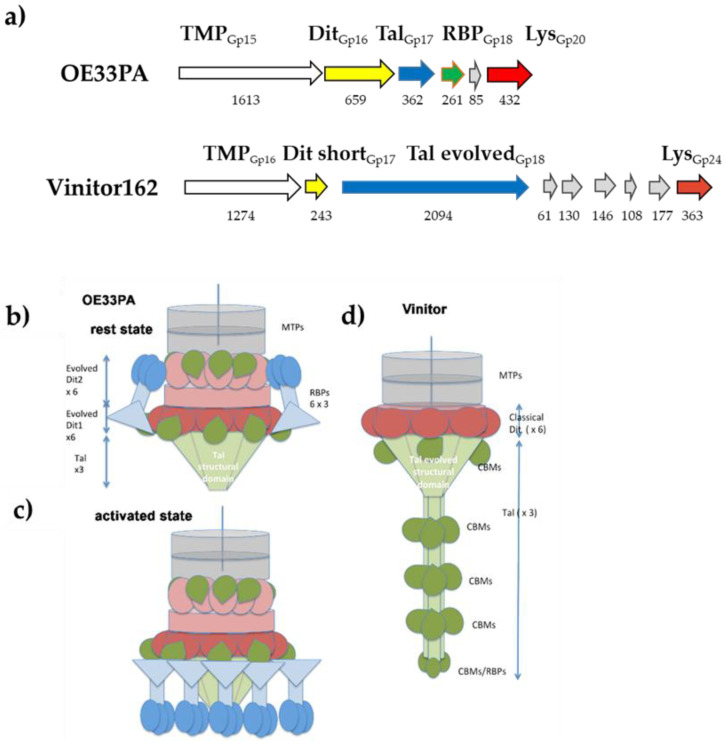
Topology models of phages OE33PA and Vinitor162. (**a**) comparison of the phage genomes between the *tmp* and *endolysin* genes (the sizes of deduced proteins in amino acids are given); (**b**,**c**) topology models of the OE33PA baseplate in resting and activated states (based on phage p2 baseplate structures [43,44]. Phages with an activation mechanism for host binding possess two distal tail (Dit) hexamers, (Dit1 in dark pink and Dit2 in light pink). Each evolved Dit monomer harbors one (or two) carbohydrate-binding modules (CBMs) (one CBM is shown here). The trimeric Tal (light green) is reduced to a mere structural domain. The trimeric receptor-binding protein (RBP) assembles shoulder and neck domains (light blue) followed by the receptor-binding domain (dark blue). The RBPs rotate by ~180° during the activation process; (**d**) Topology model of Vinitor. The classical Dit hexamer is colored dark pink. The trimeric Tal is composed for each monomer (from N- to C-terminus) of a CBM domain (dark green), a structural domain (light green) and an extended tail (light green) carrying three CBMs (dark green) and terminated by a fifth CBM/RBP domain (dark green). MTP, Major Tail Protein.

The comparative analysis of phage genomes is in agreement with these findings [23,31]. The vast majority of Siphoviruses infecting LAB possess a large organelle at their tail tip, either a bulky baseplate or an extended tail tip, bearing the RBPs and mediating host adsorption [41,42]. These essential genes are located between the *tmp* (gene coding for the Tape Measure Protein that determines the length of the tail) and the *holin/endolysin* lysis cassette (Figure 4a).

For OE33PA, HHpred [36] identified that Gp16 is an evolved distal tail (Dit) protein (the host-binding device hub docking structure), which contains carbohydrate-binding module (CBM) insertions as compared with classical Dit, related to that of *Lactococcus lactis* phage p2 (PDB ID 2WZP, Figure S3). The size of OE33PA Dit (659 amino acid residues) suggests that there should be two insertions of a CBM as previously observed in *Lactobacillus casei* phage JL1 [45,46]. However, HHpred identified only one CBM (PDB ID 4XUP) with a low probability, suggesting that the CBMs present in this Dit have folds not represented in the PDB. Gp17 was identified as a short tail-associated lysozyme (Tal) possessing only the structural domain (PDB ID 3GS9, Figure S4). The next Gp18 was identified as the RBP with a fold similar to that of the phage p2 RBP (PDB ID 1ZRU, Figure S5). These analyses strongly support that the phage OE33PA adhesion device is highly similar to that of phage p2, which recognizes saccharide-based receptors in the *L. lactis* cell wall [43,47,48]; the only difference being that its Dit is evolved, while that of phage p2 is classical. Worth noticing, several p2-like phages possess an evolved Dit [49]. Such evolved Dit may extend or complement the carbohydrate-binding activity of the RBP. In contrast to those findings, the organization of the corresponding region in the genome of Vinitor162 was strikingly different (Figure 4a). The Vinitor162 Dit protein is classical (no CBMs inserted) and the Tal is very long with a size greater than that of the TMP, which is often the longest and most easily recognizable open reading frame in *Siphoviridae*. A recent analysis of Vinitor phages revealed that their long Tal harbor four different CBMs plus a C-terminal putative RBP [23]. Our bioinformatic analysis, together with the knowledge that Dit are hexameric and Tal are trimeric within virions [42], made it possible to establish topology schemes for both phages (Figure 4b–d). This is noteworthy because if the phage OE33PA baseplate is similar to that of phage p2, it should exhibit the same activation mechanism [43,44]. As a conclusion, our analyses demonstrated the presence of different tail tip complexes in OE33PA and Vinitor, which likely governed the recognition of distinct receptors at the surface of their common host strain O. oeni IOEBS277.

### 3.6. How Do PCs Affect the Host-Killing Activity of OE33PA on O. oeni?

**Figure 5:**
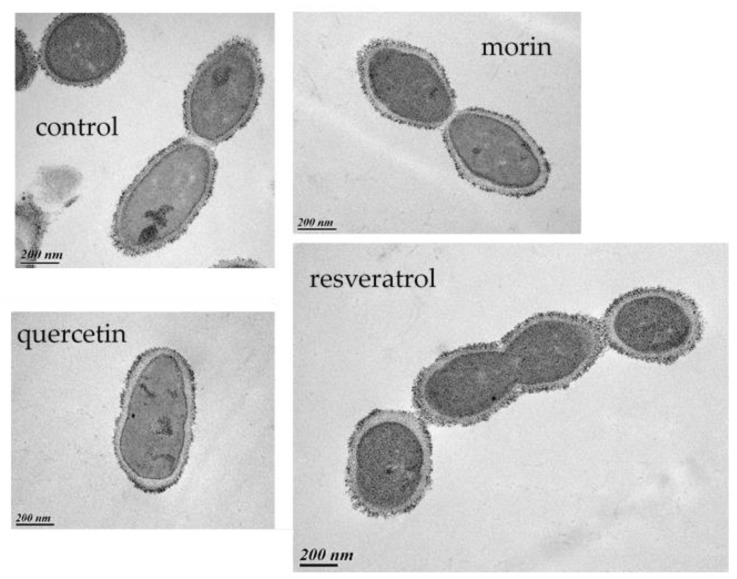
TEM observations of *O. oeni* IOEBS277 cells grown with no PCs (control), quercetin, morin or resveratrol (50 µg mL^−1^). The bars represent 200 nm.

Considering the pleiotropic effects of PCs, it is likely that they reduce the host-killing activity of OE33PA on O. oeni in a complex way, which at least involves an impairment of phage adsorption. First, we explored the possibility that PCs induce modifications of the cell envelope, which in turn may reduce phage adsorption onto the putative saccharidic cell surface receptors. Previous studies showed that O. oeni produces low levels of capsular polysaccharides in laboratory media [35]. As PCs may increase the production of polysaccharides in related LAB [17,18], we reasoned that this could mask the bacterial receptors recognized by OE33PA in *O. oeni*. We therefore assessed the presence of polysaccharides by electron microscopy following the staining of ultra-thin sections by the Thiery procedure [34]. No change in the morphology/integrity of PC-treated cells was observed. All bacterial cells produced substances that stained positively with the Thiery reaction (Figure 5) and the loosed form of the cell-linked polysaccharide layer previously described in the IOEBS277 strain was observed [35]. No significant modifications in the appearance and thickness were observed between quercetin or morin-treated cells and the controls. Similar data were observed with cells grown with resveratrol, which was shown to have no effect on phage predation.

An alternative explanation to our observations could also be that PCs may directly interact with the phage RBP (Gp18). Whether OE33PA and p2 share the same structure, a cavity would be present at the surface of the RBP, which is dedicated to the docking onto the host receptors. In OE33PA, PCs may occupy this cavity and therefore compete with adsorption on the cell wall. To explore this possibility, we first performed three additional killing-curve assays in the presence of quercetin and different concentrations of phages, yielding MOI of 0.01, 0.003 and 0.002 (Figure S6). We observed a dose-response relationship and the lower the phage concentration, the higher the inhibition of its killing effect on the host. This may indicate that when less phage particles are present, the probability for quercetin to target the cavity in the RBP increases, yielding a higher proportion of phages with reduced adhesive properties. We then made a deep analysis of the RBPs of phages p2 and OE33PA. Both proteins share 21% sequence identity and a high similarity. Moreover, only three amino acid deletions were observed between the head domains of both phages with OE33PA being the shorter (Figure S5). We could therefore readily build a homology model of the OE33PA RBP head domain (residues 160–261) based on the 3D structure of phage p2 RBP (PDB ID 1ZRU [47].

**Figure 6:**
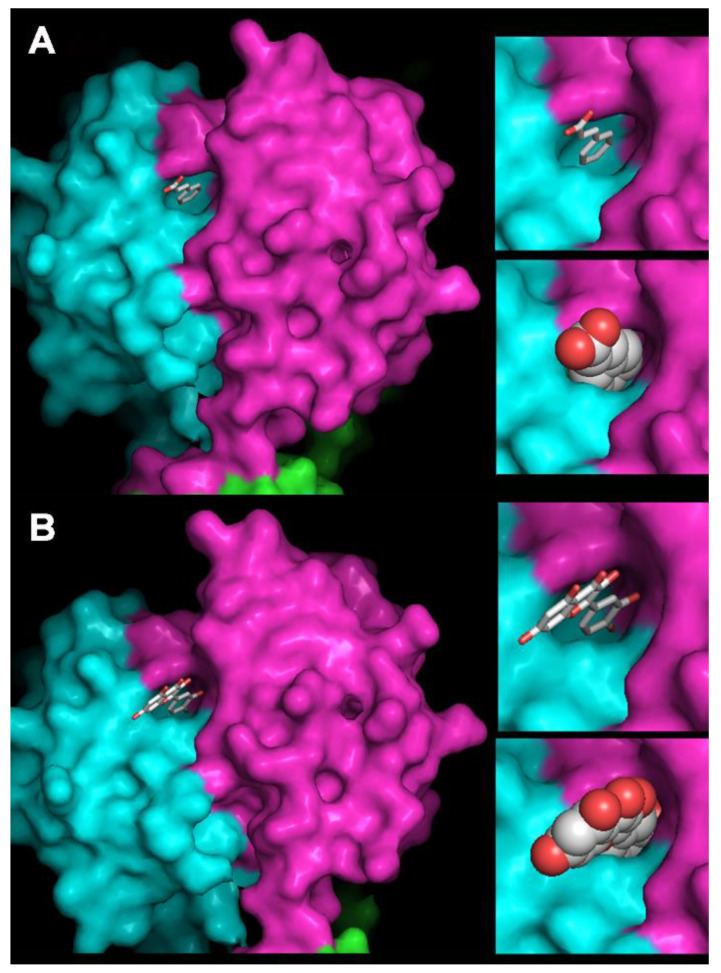
Homology model of phage OE33PA RBP with inhibitors docking. Docking of cinnamic acid (**A**) and quercetin (**B**) in the RBP binding cavity. The trimeric RBP surface is colored pink, blue and green. The binding cavity is at the interface between the pink and blue domains. Cinnamic acid and quercetin are displayed as balls and sticks in the main frame and in the upper close-up and as atomic spheres in the lower close-up. Carbons are colored white and oxygens red. Hydrogens are not displayed. Pictures made with PyMOL (The PyMOL Molecular Graphics System, Version 2.0 Schrödinger, LLC).

Phage p2 receptor-binding sites are located at the interface between two monomers of the trimeric RBP head [48]. Four key residues have been identified in phage p2 RBP for binding to occur: His 232, Asp 234, Trp 244 and Arg 256. The same residues are observed in the RBP of lactococcal phage TP901-1, with Trp replaced by a Phe amino acid [50]. In phage OE33PA RBP, Tyr 241 corresponds to p2 Trp 244 and Glu 231 to Asp 234, while p2 His 232 and Arg 256 are replaced by Gly 229 and Gly 253 in OE33PA RBP. A deep cavity was observed flanking OE33PA RBP Tyr 241 in our model (Figure 6). We fitted cinnamic acid by placing the phenyl ring in the cavity stacked against Tyr 241 with the carboxylic moiety pointing outside towards the solvent (Figure 6). Of note, there was enough space around positions R2 and R3 of cinnamic acid to accommodate its hydroxy derivatives in the cavity. A quercetin 3,4-dihydroxyphenyl ring was fitted in a position similar to that of the phenyl ring of cinnamic acid with the more hydrophylic 3,5,7-trihydroxy-4H-chromen-4-one moiety pointing towards the solvent. Further details on the interaction of both compounds within the binding site could not be safely extracted from the homology model and would be obtained only from crystal structures.

## 4. Discussion

Increasing evidence suggests that PCs are able to inhibit/increase the growth of specific microorganisms, resulting in changes of wine microbiota composition. Indeed, direct interactions between PCs and bacteria have been reported to occur and different sites of action at the cellular level have been characterized [51]. Our study on the LAB *O. oeni* brings novel aspects and more complexity into the PC-wine microbiota interplay and suggests the existence of a ménage-à-trois in which the crosstalk between phages and sensitive bacteria is finely-tuned by PCs. Indeed, the sharpness of the proposed mechanism could lie in the differential modulation of phage predation of the host as we showed that Vinitor phages, in contrast to OE33PA, were not affected by the identified PCs.

Our data are in line with previous observations showing that the infection of *O. oeni* by phage IOEB0608 (whose tail tip is similar to that of OE33PA) is less effective in a grape juice medium than in MRS broth, as judged by a 10-fold reduction in the phage titer in the supernatant [52]. The hypothesis was also raised decades ago in a study on the lytic activity of phage PL-1 on a *Lactobacillus casei* strain [19]. Even though our experiments were conducted in MRS, the proposed interactions may also occur during wine-making and add to the list of factors that explain the variability of the biomass of the *O. oeni* population and the success of MLF in wines. Indeed, several studies support this hypothesis. First, the predation of *O. oeni* by phages was shown to differ in white and red wines and this was correlated with distinct phenolic compositions of the tested wines [21,53,54]. Second, the most identified active compounds in our study were flavonoids, which represent 80–90% of the total PC content in wines with concentrations ranging from 0.5 to 300 mg L^−1^ [55]. Their proportions in wines are known to depend on the grape cultivar used, the winemaking processes that determines their extraction into the must and also the wine age.

The mechanisms by which PCs reduce the killing of *O. oeni* by phage OE33PA deserve additional work. We posit that PCs reduce the phage-cell attachment, which is well supported in the current literature on eukaryotic viruses including Coronaviruses [56,57]. Flavonoids are also known to induce fluidity changes or lipoteichoic acid release from the cytoplasmic membrane [58]. Another example shows that in the presence of quercetin (50 µg mL^−1^), *Enterococcus caccae* overexpresses many genes involved in energy production and represses genes related to stress response, translation and sugar transport [18]. Taking these data into consideration, it is likely that PCs could also act at later stages of OE33PA infection and possibly affect DNA injection and replication.

Regarding the proposed interference of PCs during the adsorption of OE33PA on *O. oeni*, we tried to consider that flavonoids and phenolic acids could target each interaction partner. No increase in the production of capsular polysaccharides on the bacterial envelope was observed by TEM. However, our data do not discard the possibility that more subtle changes may be induced by PCs in the cell envelope of *O. oeni* and other techniques and strategies are now needed to investigate the nature of the receptors recognized by OE33PA and Vinitor phages and confirm that they are different. Owing to the fact that the RBP of OE33PA and p2 in *Lc. lactis* are similar, they probably target similar structures and their complexity has been recently characterized in *Lc. lactis* [59,60]. Hence, OE33PA is likely to recognize a specific sugar moiety in the cell wall pellicle of *O. oeni.* The possible interference of PCs in the recognition of such receptors (and not that of Vinitor) still remains a working hypothesis. On the other point of view, docking analyses bring a new perspective that PCs may also target the phage tail and reduce the adsorption capacity of OE33PA, while Vinitor, which has a different tail tip, is not affected. Our docking simulations suggested that both quercetin and cinnamic acid could be considered as specific ligands of the OE33PA RBP. The presence of the bulky glucuronic acid moiety in the quercetin-O-glucoside molecule would result in the loss of interaction and supports the lack of inhibition of the compound in our experiments. Our preliminary data highlight the usefulness of docking analyses to better understand the mode of action of PCs and will be confirmed by crystallization studies [61].

As a conclusion, our data illustrate the complexity of phage-host interactions in the enological environment and the multiplicity of factors affecting the *O. oeni* population and possibly the success of MLF in wines. The proposed role of PCs in modulating phage-host interactions and the differential impact observed on OE33PA and Vinitor oenophages now open perspectives in other fields to better understand phage infection dynamics in the gut microbiota in the presence of dietary PCs [62] or develop relevant phage-based biocontrol strategies to limit plant pathogens [63].

## Figures and Tables

**Table 1:** Name and chemical structures of the hydroxycinnamic acids and flavonoids tested in killing assays with the lytic oenophages OE33PA and Vinitor.

Chemical Structure	PC Family and Functional Groups							Inhibition of Phage-Killing Activity	
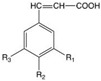	Hydroxycinnamic acids	R1	R2	R3				OE33PA	Vinitor 162
	Coumaric acid	H	OH	H				+	-
	Caffeic acid	H	OH	OH				+	-
	Cinnamic acid	H	H	H				+	-
	Flavonols	2′	3′	4′	5′	R	7		
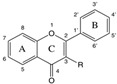	Quercetin	H	OH	OH	H	OH	OH	+	-
	Quercetin 3-O-glucuronide	H	OH	OH	H	Gluc. acid	OH	-	-
	Myricetin	H	OH	OH	OH	OH	OH	+	-
	Kaempferol	H	H	OH	H	OH	OH	-	-
	Morin	OH	H	OH	H	OH	OH	+	-
	Retusin	H	OCH3	OCH3	H	OCH3	OCH3	+	-

The intensity level reductions of phage killing activity are indicated (++, +). The absence of impact is shown (-).

## Supplementary Materials

The following materials are available online at https://www.mdpi.com/1999-4915/12/11/1316/s1, Table S1: LC-MS identification of the major phenolic compounds detected in the active fractions F9 and F11; Figure S1: The OE33PA phage lysate was incubated for 72 h without (white) or with PCs (50 µg mL^−1^) (black) in MRS_Φ_ and titrated on *O. oeni* IOEB277; Figure S2: Compared effect of quercetin on the infection cycle of OE33PA (red) or Vinitor on *O. oeni* IOEBS277; Figure S3: Analysis of Dit protein; Figure S4: Analysis of Tal protein; Figure S5: Analysis of RBP proteins; Figure S6: Impact of quercetin on the in vitro lysis assay of OE33PA against the host organism at three different MOIs.
